# Aquaporin-4, Connexin-30, and Connexin-43 as Biomarkers for Decreased Objective Sleep Quality and/or Cognition Dysfunction in Patients With Chronic Insomnia Disorder

**DOI:** 10.3389/fpsyt.2022.856867

**Published:** 2022-03-25

**Authors:** Shuai Yang, Xiao-Yi Kong, Ting Hu, Yi-Jun Ge, Xue-Yan Li, Jun-Tao Chen, Shuo He, Ping Zhang, Gui-Hai Chen

**Affiliations:** Department of Neurology (Sleep Disorders), The Affiliated Chaohu Hospital of Anhui Medical University, Hefei, China

**Keywords:** aquaporin-4, astrocyte, cognition, connexin-30, connexin-43, insomnia disorder

## Abstract

**Objectives:**

To examine serum concentrations of aquaporin-4 (AQP4), connexin-30 (CX30), connexin-43 (CX43), and their correlations with cognitive function in the patients with chronic insomnia disorder (CID).

**Methods:**

We enrolled 76 subjects with CID and 32 healthy controls (HCs). Serum levels of AQP4, CX30, and CX43 were measured by enzyme-linked immunosorbent assays. Sleep quality was assessed with the Pittsburgh Sleep Quality Index (PSQI) and polysomnography, and mood was evaluated with 17-item Hamilton Depression Rating Scale and 14-item Hamilton Anxiety Rating Scale. Cognitive function was evaluated by the Chinese-Beijing Version of Montreal Cognitive Assessment (MoCA-C) and Nine Box Maze Test.

**Results:**

The serum levels of AQP4, CX43, and CX30 were significantly reduced in the CID group compared to the HCs. Partial correlation analysis showed that the biomarkers showed no significant correlations with PSQI score, AHI, ODI and TS90, but AQP4, CX43, and CX30 were positively associated with the percentage and total time of slow wave sleep in the CID group. Serum concentrations of AQP4 and CX30 were positively associated with MoCA-C score in the CID group, and AQP4 level negatively correlated with spatial working memory errors.

**Conclusions:**

Subjects with CID patients have decreased serum levels of AQP4, CX30, and CX43 indicating astrocyte dysfunction, which could be related to poor objective sleep quality and/or cognition dysfunction.

## Introduction

Insomnia is characterized by difficulty in initiating and/or maintaining sleep with unintentional early morning awakenings along with significant impairment of daytime functioning (1). Chronic insomnia disorder (CID) is a heavy burden to individuals and society as it is associated with physical and mental diseases (2–4). Despite recent progress in the treatment of chronic insomnia, many patients still struggle with this condition (5). One reason is that the mechanism underlying CID remains unclear.

Whether CID is a result of subtle brain injury remains a topic of intense debate. A correlation between brain injury and insomnia is supported by imaging findings showing that CID patients have some degree of brain damage (6, 7). Magnetic resonance imaging revealed atrophy in frontal and subcortical areas and changes in the white matter tracts connecting these areas in patients with CID compared to healthy controls (HCs) (8). Another group proposed that insomnia is related to brain functional connectivity abnormalities (9). Unfortunately, neuroimaging study reproducibility has been increasingly questioned due to low test-retest reliability, and identifying additional biomarkers is of particular interest to researchers and clinicians (10). Serological testing is a promising option because of the simplicity and convenience. In addition, past studies have mainly shown a relationship between insomnia and specific brain regions (11). Few researchers have focused on changes in more minute structures, such as at the cellular level.

Although astrocytes outnumber neurons, it is thought that astrocytes only perform a nutritional and supportive function in the brain. In 1895, Cajal proposed that astrocytes regulate sleep by extending their processes into synapses during sleep and retracting these processes during wakefulness (12). However, it was not until 2009 that the first evidence show that the sleep/wake cycle was regulated by these glial cells (13). Since then, the importance of astrocytes in sleep regulation has become increasingly apparent. It is widely known that brain damage is associated with cognitive impairment (14). While cognition is traditionally attributed to neuronal activity, it is becoming increasingly clear that astrocytes also contribute to cognitive function and sleep regulation (15). Thus, it is necessary to conduct more studies to explore correlations between astrocyte function and the states of cognition and sleep.

We previously showed that changes in levels of neurofilament heavy and light chains, neuron-specific enolase, and S100 calcium binding protein B (S100B), which reflect structural damage in neurons and astrocytes, were correlated with insomnia severity and/or cognitive impairment (16). More importantly, even after long-term effective treatment, the serum S100B levels did not significantly restore to normal concentration in the patients with CID (16). Thus, the relief/remission of their illness may not be accompanied by normalization of changed protein levels (damage of astrocytes structure may be a trait) (16). Subjects with CID also exhibit significantly lower levels of glial cell line-derived neurotrophic factor (GDNF) and brain-derived neurotrophic factor (BDNF) than healthy people (17). These findings strongly implicate astrocyte dysfunction in CID, but more evidence is needed to support this hypothesis (17). Additional studies are needed to investigate other astrocyte-related markers to better understand the mechanism of CID.

Previous studies typically focused astrocytes themselves rather than their networks. However, one of the unique characteristics of astrocytes is the high level of connexins including connexin-30 and -43 (CX30 and CX43) (18). Connexins form gap-junction channels that are responsible for direct intercellular communication and allow small molecules and ions to pass freely between cells (19). Recently, increased Cx43 levels were found during recovery sleep in adult male Wistar rats that previously experienced sleep deprivation (20). Cx30 and Cx43 mRNA show time-of-day-dependent expression in the suprachiasmatic nucleus of the hypothalamus, which is believed to play an important role in sleep-wake regulation (21). These results suggested that astrocyte connexins contribute to sleep regulation. Another major function of sleep is to clear out metabolic waste products from the brain through the glymphatic system (22). Aquaporin-4 (AQP4) is mainly expressed on astrocytic end-feet, which play a significant role in waste clearance (23). Previous findings revealed that AQP4 deletion impaired glymphatic transport and short-term memory after chronic sleep deprivation in mice (24). However, there is no research describing a link between AQP4 and CID or investigating whether serum AQP4 levels are associated with cognitive function in subjects with CID.

AQP4 and CXs have been investigated in neurological diseases such as Parkinson's disease, hypoxic brain edema, and traumatic brain injury (25–28). For example, Ramiro et al. found that serum AQP4 levels correlated with stroke severity and predicted early neurological function improvement in stroke patients (29). Swelling of astrocytic end-foot processes is accompanied by decreased cell membrane expression of AQP4 and CXs (30, 31). Protein level alterations in the brain may be in part reflected in the blood, but the precise source of serum AQP4 and CXs has not been clarified (32). One possible explanation is the presence of brain-derived vesicles containing AQP4 and CXs. When astrocyte function decreases, fewer brain-derived vesicles containing AQP4 and CXs are released, leading to lower serum concentrations.

The aim of this study was to investigate the possibility of astrocyte dysfunction in CID, we measured serum levels of AQP4, CX43, and CX30 in subjects with CID and HCs. We also explored whether altered serum levels of these astrocyte-related markers correlated with poor sleep quality and/or cognitive impairment, and if they could be used to distinguish between the CID and HC groups.

## Materials and Methods

### Subjects

**Figure 1:**
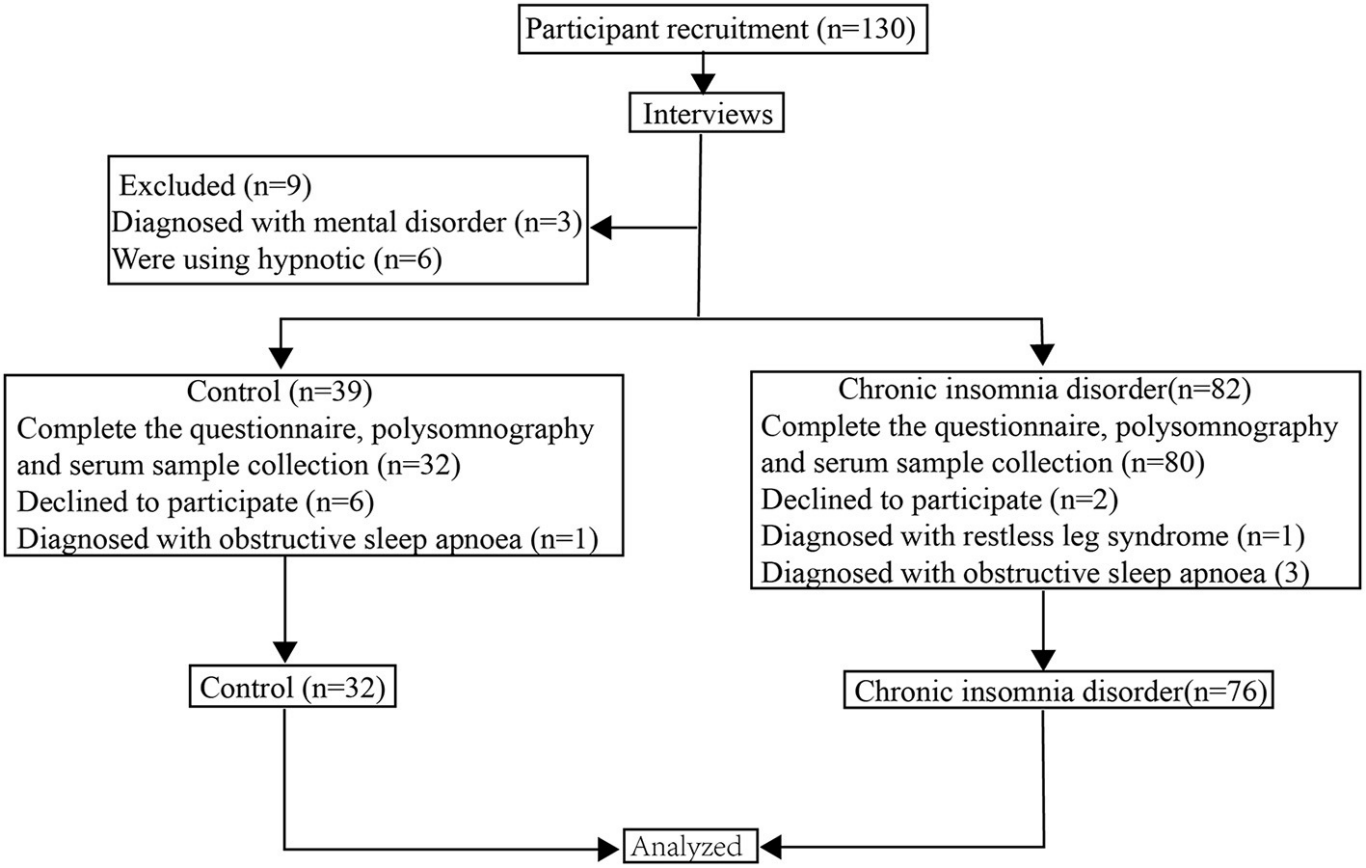
The flowchart of the study participants.

A total of 108 people, including 76 subjects with CID and 32 HCs, were involved in the study. The flowchart of the study participants is presented in Figure 1. All subjects were recruited from the Clinic of Sleep Disorders in the Affiliated Chaohu Hospital of Anhui Medical University. The patients (30 males and 46 females between 18 and 60 years old) were diagnosed with CID following the criteria in the third edition of the International Classification of Sleep Disorders (33). Furthermore, they should meet the following criteria: (1) age is 18–60 years old; (2) normal ability to complete the assessment scales and the memory test without problem in hearing and vision; (3) not experiencing any somatic disease (including immunologic, endocrine, cardiovascular, neurologic, liver, or kidney disease or organic brain disease) or any other psychiatric disorders; (4) not taking sedatives, antidepressants, antipsychotics, or any other drugs within 4 weeks; (5) non-pregnant or non-lactating females.

Thirty-two HCs were chosen as the control group using the following criteria: (a) no complaints or history of insomnia or mood disorders, (b) total score <7 on the Pittsburgh Sleep Quality Index (PSQI), (c) total score <7 on the Hamilton Depression Rating Scale-17 (HAMD), (d) a total score <7 on the Hamilton Anxiety Scale-14 (HAMA), and (e) Montreal Cognitive Assessment, Chinese-Beijing Version (MoCA-C) score >26 (34–37). Control subjects were recruited from the physical examination centers at the same hospitals. They received a comprehensive medical involving a medical history review, electrocardiogram, blood testing (hematology and serology), and physical examination. The control subjects were also matched to cases by age, gender, geographic region, and ethnicity. As pre-established criteria, any somatic disease (including immunologic, endocrine, cardiovascular, neurologic, liver, or kidney disease or organic brain disease) or any other psychiatric disorders were excluded.

### Baseline Data Collection

Information on demographic characteristics, medical history, and family history were collected using a questionnaire developed by our group.

### Assessment of Mood States

Depression and anxiety severity were separately assessed with the HAMD and HAMA, respectively (35, 36). The maximum score on the HAMD is 52, and the highest score on the HAMA is 56. A higher total score indicates greater depression or anxiety symptom severity. In China, the HAMD score cut-offs are as follows: ≤ 7, no depression; ≥8, mild depression; ≥18, moderate depression; and ≥24, severe depression. HAMA ≥7 indicates that the patient may have anxiety, and HAMA <7 indicates that the patient has no anxiety.

### Subjective and Objective Sleep Evaluations

The PSQI is designed to measure subjective quality of sleep over the past month. It measures seven components of sleep: latency, quality, duration, disturbances, efficiency, the use of sleep medications, and daytime dysfunction. A higher total score indicates worse sleep quality (34).

All participants underwent one night of polysomnography (PSG) to acclimatize to the novel environment followed by 2 nights of PSG to collect study data. The recording assessed sleep continuity and structure. The former was measured as total sleep time (TST), sleep efficiency (SE), and sleep onset latency (SOL); the latter was evaluated for the following parameters: percentages of sleep stage 1 (N1%) and slow wave sleep (SWS, N3%), time awake after sleep onset, and time (REM) and percentage (REM%) of rapid eye movement sleep, apnea hypopnea index (AHI), oxygen desaturation index (ODI), SpO2 time <90% (TS90). Sleep parameters were staged according to the 2012 American Academy of Sleep Medicine Manual for the Scoring of Sleep and Associated Events recommendations (37).

### Evaluation of Cognitive Function

Cognitive function was evaluated by experienced clinicians using MoCA-C and a modified version of the Nine Box Maze Test (NBMT). The MoCA-C scale is used for a screening of general cognitive abilities. The maximum score for the MoCA-C is thirty, however, the score <26 indicates the cognitive impairment in China (38). A modified version of the Nine Box Maze Test (NBMT) was used to evaluate memory, including spatial/object working memories (SWM/OWM), spatial/object reference memories (SRM/ORM), and object recognition memory (ORcM) (39). The testing procedures are described in detail elsewhere (40). The numbers of errors were counted to examine SWM, OWM, SRM, ORM, and ORcM performance.

### Serum Sample Collection

Serum from blood samples was obtained the morning following PSG and immediately stored at −80°C until analysis. Serum concentrations of AQP4, CX43, and CX30 were measured by quantitative sandwich enzyme-linked immunosorbent assays according to the manufacturer's instructions (Wuhan colorfulGene biological technology, Wuhan, China).

### Statistical Analysis

All data were analyzed using SPSS^®^ 20.0 for Windows (IBM Corp., Armonk, NY, USA). Normally distributed data are expressed as mean ± standard deviation (SD). Mann-Whitney *U*-tests were performed to analyze non-parametric data expressed as the 25th, 50th, and 75th percentiles (P25, P50, and P75, respectively). Partial correlation analyses were used to analyze correlations between serum biomarkers and sleep parameters and PSQI (adjusted for sex, age, education, HAMD, and HAMA) and between serum biomarkers and general cognitive function and multi-dimensional memory (adjusted for sex, age, education, HAMD, HAMA, and PSQI). The diagnostic accuracies of biomarkers were evaluated by constructing receiver operating characteristic (ROC) curves and calculating the areas under the curve (AUCs). Differences were considered significant at *P* < 0.05 for all analyses.

## Results

### General Information and Sleep Parameters

There were no significant differences in sex, age, or education between the two groups (*Ps* > 0.05, Table 1). Compared to the HC group, patients with CID had significantly higher HAMD and HAMA scores (*Ps* < 0.001, Table 1). Meanwhile, the patients with CID had significantly longer SOL and N1, and significantly lower SE, TST, and N3 than the HC (*Ps* < 0.001, Table 2). There were no significant differences in AHI, ODI, and TS90 between the two groups (*Ps* > 0.05, Table 2). These results indicated that the patients who were recruited into the study met our inclusion criteria. Sleep parameters are detailed in Table 2.

**Table 1:** Demographic data and HAMD and HAMA scores.

**Terms**	**CID**	**HC**	**Statistics**	***P***
Number of cases	76	32		
Male/female	30/46	13/19	*c*^2^ = 0.012	0.911
Age (years)	49 [40,53]	45 [32, 57]	*Z* = −0.131	0.896
Education (years)	9 [6,12]	9 [6,12]	*Z* = −0.464	0.643
HAMD (score)	8 [6,10]	2 [1,5]	*Z* = −7.006	<0.001
HAMA (score)	9 [7,13]	3 [1,5]	*Z* = −7.556	<0.001

*Normal distribution variables (Mean ± SD); non-normal distribution variables (P50 [P25, P75])*.

*CID, chronic insomnia disorder; HAMA, Hamilton Anxiety Scale (14 items); HAMD, Hamilton Depression Rating Scale (17 items); HC, healthy control*.

**Table 2:** Sleep quality and respiratory parameters.

**Categories**	**Terms**	**CID (*n* = 76)**	**HC (*n* = 32)**	**Statistics**	***P***
Subjective sleep	PSQI (score)	16 [15,17]	4 [3,6]	*Z* = −8.214	<0.001
Objective sleep	TST (min)	375.0 ± 85.0	448.0 ± 48.0	*t* = 4.56	<0.001
	SOL (min)	25.7 [14.0, 49.9]	8.5[5.0, 17.5]	*Z* = −4.32	<0.001
	SE (%)	72.3 ± 14.6	86.8 ± 11.5	*t* = 4.78	<0.001
	N1	114.5[67.0, 175.4]	47.5 [33.9, 61.0]	*Z* = −6.12	<0.001
	N1%	29.5 [21.0, 46.9]	10.1 [7.7, 14.0]	*Z* = −7.10	<0.001
	N3	34.2 ± 26.8	83.0 ± 43.1	*t* = 7.07	<0.001
	N3%	9.6 ± 7.4	18.4 ± 8.7	*t* = 5.27	<0.001
	REM	39.0 ± 26.3	83.5 ± 23.6	*t* = 8.31	<0.001
	REM%	9.0 [5.3, 15.5]	18.6 [16.1, 22.7]	*Z* = −5.47	<0.001
Respiratory parameters	AHI	0.6 [0.1, 1.7]	0.1 [0.0, 1.7]	*Z* = −1.88	0.060
	ODI	0.7 [0.1, 1.8]	0.1 [0.3, 1.1]	*Z* = −1.29	0.196
	T90	0.0 [0.0, 2.2]	0.0 [0.0, 2.0]	*Z* = −0.50	0.615

*Normal distribution variables (Mean ± SD); non-normal distribution variables (P50 [P25, P75])*.

*AHI, apnea hypopnea index; CID, chronic insomnia disorder; HC, healthy control; N1, sleep stage 1; N1%, percentage of the sleep stage 1; N1, sleep stage 1; N3%, percentage of the sleep stage 3; ODI, oxygen desaturation index; PSQI, Pittsburgh Sleep Quality Index; REM, rapid eye movement sleep time; REM%, percentage of the rapid eye movement sleep time; SE, sleep efficiency; SOL, sleep onset latency; TST, total sleep time; TS90, SpO2 time <90%*.

### Cognitive Function and Serum Biomarkers in CID

**Figure 2:**
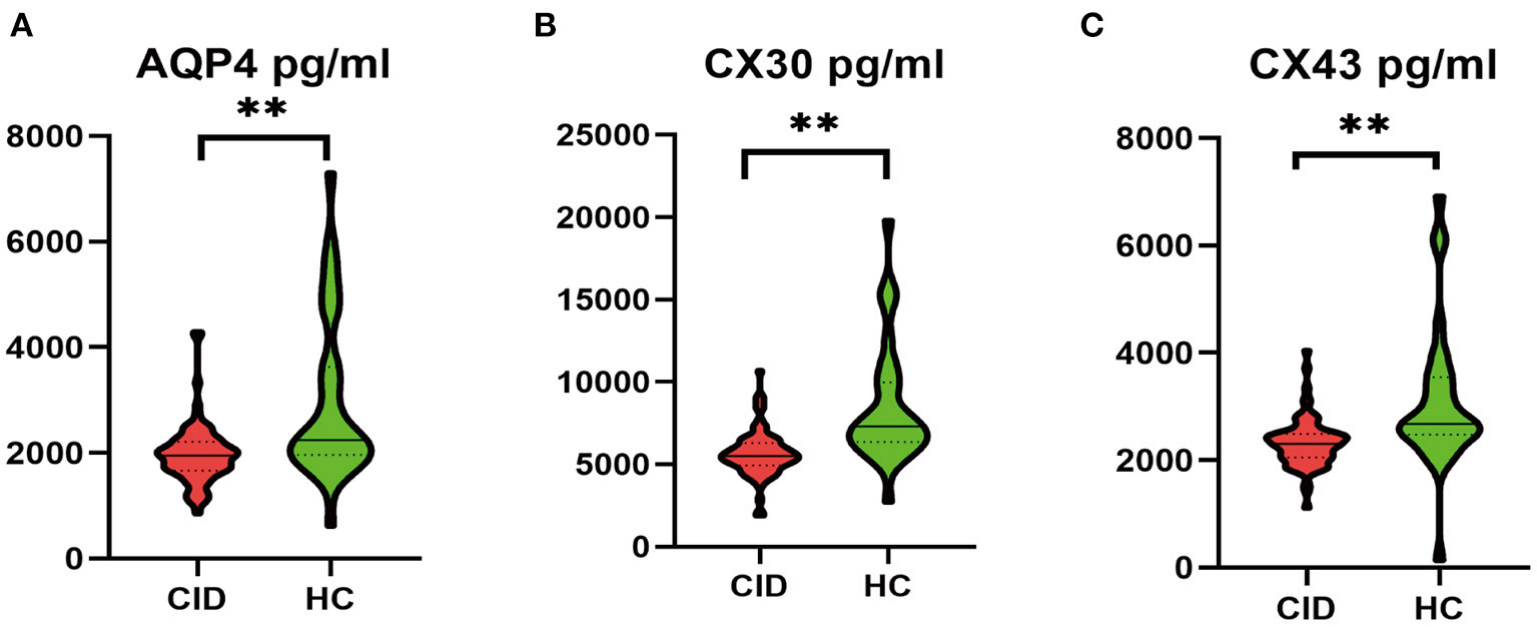
Comparison of serum protein levels between CID patients and HCs. **P < 0.01. AQP4, aquaporin-4; CID, chronic insomnia disorder; CX30, connexin 30; CX43, connexin 43; HC, healthy control.

Patients with CID had significantly lower MoCA-C scores (*P* < 0.001) and more SWM (*P* < 0.001) and ORcM (*P* < 0.01) errors than HC (Table 3). CID patients had significantly lower serum levels of AQP4, CX43, and CX30 (*Ps* < 0.001) compared to the HC group (Figure 2).

**Table 3:** Cognitive performance.

**Terms**	**CID (*n* = 76)**	**HC (*n* = 32)**	***P***
MoCA-C (score)	25 (22, 26)	27 (25, 28)	<0.001
ORM (number of errors)	0 (0, 0)	0 (0, 0)	0.618
SRM (number of errors)	0 (0, 0)	0 (0, 0)	0.205
OWM (number of errors)	1 (0, 1)	0 (0, 1)	0.066
SWM (number of errors)	2 (1, 4)	1 (0, 2)	<0.001
ORcM (number of errors)	0 (0, 1)	0 (0, 0)	<0.01

*Normal distribution variables (Mean ± SD)*.

*CID, chronic insomnia disorder; HC, healthy control; MoCA-C, Chinese-Beijing Version of Montreal Cognitive Assessment; ORcM, object recognition memory; ORM, object reference memory; OWM, object working memory; SRM, spatial reference memory; SWM, spatial working memory*.

### Correlations Between Biomarkers, Sleep Parameters, and Cognitive Performance in CID

Partial correlations (adjusted for age, sex, education HAMD, and HAMA) between serum biomarkers and objective sleep parameters are listed in Table 4. The serum concentrations of AQP4, CX43, and CX30 were positively correlated with N3 (*r* = 0.250, *P* < 0.05; *r* = 0.323, *P* < 0.01; *r* = 0.334, *P* < 0.01). The same results were also observed in N3% (*r* = 0.233, *P* < 0.05; *r* = 0.302, *P* < 0.05; *r* = 0.335, *P* < 0.01). There were no significant correlations between serum astrocyte-related marker levels and PSQI scores, AHI, ODI, and TS90 (*Ps* >0.05). Partial correlation analysis (adjusted for age, sex, PSQI, HAMD, HAMA, and education) indicated that serum AQP4 level was positively correlated with MoCA-C score (*r* = 0.357, *P* < 0.01) and negatively correlated with SWM errors (*r* = −0.435, *P* < 0.01; Table 5). CX30 level was negatively correlated with MoCA-C score (*r* = 0.2.71, *P* < 0.01; Table 5).

**Table 4:** Partial correlation analysis between AQP4/CX43/CX30 and sleep quality.

**Terms**	**AQP4 (pg/mL)**	**CX43 (pg/mL)**	**CX30 (pg/mL)**
PSQI	0.046	−0.095	−0.101
TST	−0.053	−0.114	−0.085
SOL	−0.103	−0.003	−0.146
SE	−0.028	−0.077	−0.062
N1	−0.132	−0.206	−0.137
N1%	−0.095	−0.187	−0.132
N3	0.250^*^	0.323^**^	0.334^**^
N3%	0.233^*^	0.302^*^	0.335^**^
REM	−0.017	0.068	0.095
REM%	−0.024	0.062	0.098
AHI	−0.075	−0.204	−0.128
ODI	−0.049	−0.178	−0.097
TS90	−0.036	−0.158	−0.074

*Controlling for age, sex, education, HAMD, and HAMA*.

* *P < 0.05 and*

** *P < 0.01*.

*AHI, apnea hypopnea index; AQP4, aquaporin-4; CX30, connexin 30; CX43, connexin 43; N1, sleep stage 1; N1%, percentage of the sleep stage 1; N3, sleep stage 3; N3%, percentage of the sleep stage 3; ODI, oxygen desaturation index; PSQI, Pittsburgh Sleep Quality Index; REM, rapid eye movement sleep time; REM%, percentage of the rapid eye movement sleep time SE, sleep efficiency; SOL, sleep onset latency; TST, total sleep time. TS90, SpO2 time <90%*.

**Table 5:** Partial correlation analysis between AQP4/CX43/CX30 and cognitive performance.

**Terms**	**Cognitive performance**					
	**MoCA-C**	**ORM**	**SRM**	**OWM**	**SWM**	**ORcM**
AQP4 (pg/mL)	0.357^**^	0.029	−0.031	−0.080	−0.435^**^	−0.188
CX43 (pg/mL)	0.202	−0.081	−0.068	0.111	−0.112	0.042
CX30 (pg/mL)	0.271^*^	−0.010	−0.190	−0.143	−0.210	0.001

*Controlling for age, sex, education, PSQI, HAMD, and HAMA*.

* *P < 0.05 and*

** *P < 0.01*.

*AQP4, aquaporin-4; CX30, connexin 30; CX43, connexin 43; MoCA-C, Chinese-Beijing Version of Montreal Cognitive Assessment; ORcM, object recognition memory; ORM, object reference memory; OWM, object working memory; SRM, spatial reference memory; SWM, spatial working memory*.

## Discussion

### CID Is Associated With Astrocyte Dysfunction

In present study, we found that serum AQP4, CX43, and CX30 levels were significantly lower in the CID group compared to healthy individuals. These results indicate a certain extent of astrocytes dysfunction in patients with CID.

Our group has spent several years exploring the correlation between insomnia and astrocytes (16, 17). We found altered serum levels of astrocyte-related biomarkers in CID patients, with increases in S100B and glial fibrillary acidic protein and decreases in BDNF and GDNF, which led us to hypothesize a role of astrocyte dysfunction in CID (14). Interestingly, serum S100B levels did not normalize after effective therapy, indicating that astrocyte dysfunction may be an inherent trait in subjects who develop CID (13). However, this evidence is still preliminary (16). To obtain more results to support this hypothesis, we evaluated another group of astrocyte-related markers: AQP4, CX43, and CX30.

AQP4 and CXs are mainly expressed in astrocytes in the brain. We are aware of only one study describing the levels of AQP4 in the blood in neurological disorders, suggesting the lower the serum AQP4 levels, the higher the number of cerebral microbleed evaluated by MRI in the patients with Intracerebral Hemorrhage (32). This indicated an association between serum AQP4 levels and brain structure damage to some extent. Moreover, astrocytes dysfunction is found in most of brain pathologies, and are associated with changes in CX expression and functions. The basal swelling of astrocytic end-foot processes is accompanied by decreased expression of CX at the membrane (31). Obstructive sleep apnea (OSA) commonly co-occurred with insomnia and related to cognitive problems (41, 42). In addition, a recent study showed an increase in astrocytic AQP4 levels in mice, following exposure to chronic intermittent hypoxia (one of the main features of OSA) (43). In this study, none of the patients had OSA based on the respiratory PSG parameters. Thus, we have reason to believe that patients with CID have some astrocytes dysfunction according to the current study.

### Astrocyte-Related Markers Were Associated With Sleep Homeostasis

Astrocytes are able to detect and respond to neuronal activity by increasing local calcium (Ca^2+^) transients. These astrocytes communicate through CXs and release gliotransmitters to modulate neuronal activity at multiple locations (44). Therefore, astrocytes are ideally positioned to promote synchronization of large-scale neuronal networks, such as slow wave sleep (SWS), which is a reliable index of sleep homeostasis (45). The role of astrocytes in sleep homeostasis was previously investigated in the dnSNARE (dominant-negative soluble N-ethylmaleimide-sensitive fusion protein attachment protein receptor) mouse model. The authors concluded that defective astrocyte transmission could reduce SWS but did not influence REM sleep or awakening (13). Others reported that blockade of astrocytic CX43 also reduced slow wave amplitude in mice (46). Moreover, the study found that AQP4 was also linked with SWS (22). However, previous reports were mainly based on animal experiments, and this association has not yet been established in CID patients. In the current study, AQP4, CX43, and CX30 levels in subjects with CID all positively correlated with N3 sleep, also known as SWS. Our results support the idea that astrocytes may play an important role in sleep homeostasis. Interestingly, there were no significant correlations between serum astrocyte-related marker levels (AQP4, CX43, and CX30) and subjective sleep parameters (PSQI scores). This suggests that subjective and objective sleep appear to reflect two distinct neurophysiological processes.

### Astrocyte-Related Markers Were Associated With Cognitive Impairment

Astrocytes play a significant role in cognitive abilities. They are capable of (1) regulating extracellular glutamate and potassium ion uptake as well as Ca^2+^ concentration, (2) releasing and taking up neurotransmitters, and (3) controlling energy supply to neurons to facilitate cognition (47). Indeed, a recent study find that AQP4 knockout mice exhibited impaired memory consolidation in the Morris Water Maze used to test learning and memory function (48). In addition, microinjection of TAT-Gap19 (a Cx43-specific hemichannel inhibitor) into the ventricle of mice impaired short-term spatial memory in a delayed spontaneous alternation Y-maze task (49). One study also showed that mice lacking CX30 have impaired memory (50). However, it is not clear whether astrocyte dysfunction is associated with cognitive dysfunction in patients with CID. Our findings showed that serum CX30 and AQP4 levels positively correlated with cognition assessed by MoCA-C and NBMT in patients with CID. Contrary to expectations, this study did not reveal a correlation between CX43 and cognitive impairment. This may be due to the high molecular weight of CX43, which cannot easily pass the blood-brain barrier (19). It is unfortunate that we were only able to assess blood samples; future investigations can expand our findings by measuring AQP4 and CX levels in cerebrospinal fluid (CSF). It is notable that lower AQP4 expression was also observed in CSF from patients with Alzheimer's disease and Optic Neuromyelitis (51, 52). In addition, recent studies also demonstrated that the distribution of cerebrospinal fluid appears to be under a circadian control and that AQP4 seems to support this rhythm (53).

### Possible Diagnostic and Prognostic Value of Astrocyte-Related Markers

ROC analysis was conducted to assess these biomarkers' discriminatory power for CID. The results showed that three proteins had AUC values of 0.726–0.823. The optimum cut-off values for serum AQP4, CX43, and CX30 for identifying subjects with CID were 2109.7, 2542.7, and 6348.5 pg/mL, respectively (Table 6). The use of biomarkers as an objective index may help support making a diagnosis of CID. Combining these three indicators may provide a sensitive and specific approach to distinguish between the CID and healthy people.

**Table 6:** Characteristics of potential blood biomarkers for CID in ROC analysis.

	**AUC**	**Cut-off (pg/mL)**	**Sensitivity**	**Specificity**	**95% CI**
AQP4	0.726	≤ 2109.7	0.711	0.625	(0.618, 0.833)
CX43	0.772	≤ 2542.7	0.816	0.719	(0.668, 0.876)
CX30	0.823	≤ 6348.5	0.776	0.781	(0.733, 0.914)

*AQP4, aquaporin-4; AUC, area under the curve; CI, confidence interval; CID, chronic insomnia disorder; CX30, connexin 30; CX43, connexin 43; ROC, receiver operating characteristic*.

## Limitations

Our findings should be considered in the context of several limitations. Our study is limited by its cross-sectional study design, we did follow up subjects with CID to assess patient outcomes. Therefore, it cannot be determined whether astrocyte-related markers are causally related to CID pathogenesis. In addition, the sample size was too small to accurately evaluate the different contributions of each biomarker to CID, and the results need to be validated in a larger sample. Finally, our research was limited to peripheral blood; future investigations should be performed with CSF samples.

## Conclusions

The patients with CID had decreased concentrations of serum AQP4, CX43, and CX30, suggesting astrocytic pathological damages, which were linked to the decline of objective sleep quality and/or cognition ability.

## Data Availability Statement

The raw data supporting the conclusions of this article will be made available by the authors, without undue reservation.

## Ethics Statement

The studies involving human participants were reviewed and approved by Ethics Committee of The Affiliated Chaohu Hospitals of Anhui Medical University approved the study (Number KYXM-202108-005). The patients/participants provided their written informed consent to participate in this study.

## Author Contributions

G-HC: involved in the concept generation, study design, application for funding and ethical committee, conduction of the study, and data acquisition. SY and PZ: data acquisition, analysis and interpretation of the PSG data, data entry, statistical analysis and interpretation, and preparation of the working draft of the manuscript. TH and X-YK: data acquisition. Y-JG, X-YL, J-TC, and SH: data acquisition, storage, and retrieval. All authors approved the final version of the manuscript prior to the submission.

## Funding

This work was financially supported by the National Natural Science Foundation of China (81671316) and Anhui Medical University Foundation (2020xkj053).

## Conflict of Interest

The authors declare that the research was conducted in the absence of any commercial or financial relationships that could be construed as a potential conflict of interest.

## Publisher's Note

All claims expressed in this article are solely those of the authors and do not necessarily represent those of their affiliated organizations, or those of the publisher, the editors and the reviewers. Any product that may be evaluated in this article, or claim that may be made by its manufacturer, is not guaranteed or endorsed by the publisher.
